# Vaccination in Germany

**Published:** 1882-10

**Authors:** 


### OFFICIAL LETTER OF THE FRENCH AMBASSADOR AT BERLIN TO THE SECRETARY OF PUBLIC INSTRUCTION AT PARIS.

*To the President of the Council:*—By a letter dated September 27, 1880, the ambassador had sent a series of documents to the department regarding vaccination in Germany. As a supplement to those communications, I think the following statements will give complete information. The German press has discussed for some time the discoveries of two German physicians, Pissin and Reisner, in regard to proceedings which allow the animal virus (cowpox) to be kept a certain length of time without losing its efficiency. According to the statement of the journals, the health office of the German Empire has approved the efficiency of these methods, and the possibility of substituting a similar improvement on human virus as is done already in Hesse. The human virus prepared after this method is tenable, easy to preserve and transportable, and it has been introduced into general practice, notwithstanding the serious inconveniences which result from transmission of contagious diseases. Till now, the attention has been directed only to syphilis, but the discoveries of a German savant, Dr. Koch, have greatly enlarged the field of observations. Dr. Koch has scientifically proved the identity of origin of scrofula and tuberculosis. He has found in the human blood a microscopic germ susceptible of dimorphism. This germ is able to produce either of these diseases, according to its development. The dangers having been revealed by Dr. Koch, the methods of Pissin and Reissner have called further attention to this point in Germany, where vaccination is obligatory. According to a law of

April 4, 1874, every child has to be vaccinated before the age of two years, and revaccinated before it is twelve years of age. In cases of unsuccessful vaccination the operation is repeated the next year. The law makes all persons responsible, and fixes certain penalties for remonstrances against it. The statistical tables prove the efficacy and the good results of this law. Germany has been free from epidemics of variola, which gathers so many victims in the large cities of the neighboring states, as St. Petersburg, Warsaw, Vienna and Prague. The immunity stated in the principal German cities, as Berlin, Munich, Hamburg, Breslau, extends also to the ports, as Bremen, Altona, Stettin, Danzig, which latter are in regular contact with the infected countries. It is further observed that variola ceases entirely to make its appearance, and there are but isolated cases in Prussia, Silesia and Posen, which are bordering the Russian provinces, where variola is always epidemic. There is formed, thanks to obligatory vaccination, a veritable healthy frontier.

(Signed) DE COURCEL.

—*Bulletin de l' Académie de Médecine.*

